# Redesigning a Sentinel Surveillance System for Collecting and Disseminating Near Real-Time Agricultural Injury Reports: System Usability Study

**DOI:** 10.2196/13621

**Published:** 2019-08-02

**Authors:** Bryan Weichelt, Tomi Heimonen, Serap Gorucu, Emily Redmond, Josef Vechinski, Kurt Pflughoeft, Casper Bendixsen, Marsha Salzwedel, Erika Scott, Kang Namkoong, Mark Purschwitz, Risto Rautiainen, Dennis J Murphy

**Affiliations:** 1 National Farm Medicine Center Marshfield Clinic Research Institute Marshfield, WI United States; 2 Department of Computing and New Media Technologies University of Wisconsin-Stevens Point Stevens Point, WI United States; 3 Department of Agricultural and Biological Engineering Penn State University University Park, PA United States; 4 Marshfield Clinic Information Services Marshfield Clinic Health System Marshfield, WI United States; 5 Northeast Center for Occupational Health and Safety Bassett Medical Center Cooperstown, NY United States; 6 Department of Communication University of Maryland College Park, MD United States; 7 Department of Biosystems and Agricultural Engineering University of Kentucky Lexington, KY United States; 8 Central States Center for Agricultural Safety and Health University of Nebraska Medical Center Omaha, NE United States

**Keywords:** agriculture, risk, wounds and injuries, safety, farms, news, newspaper article

## Abstract

**Background:**

Injury data and reports provide valuable information for both public and private organizations to guide programming, policy, and prevention, but in the increasingly complex and dangerous industry of US agriculture, the injury surveillance needed to produce this data is lacking. To address the gap, AgInjuryNews was established in 2015. The system includes fatal and nonfatal injury cases derived from publicly available reports, including occupational and nonoccupational injuries, occurring in the agricultural, forestry, and fishing (AFF) industry.

**Objective:**

The study aimed to develop a stakeholder-engaged redesign of the interactive, up-to-date, and publicly available dataset of US AFF injury and fatality reports.

**Methods:**

Instructor-led heuristic evaluations within a 15-student undergraduate course, data from 8 student participants of laboratory-based usability testing and 2016 and 2017 AgInjuryNews-registered user surveys, coupled with input from the National Steering Committee informed the development priorities for 2018. An interdisciplinary team employed an agile methodology of 2-week sprints developing in ASP.NET and Structured Query Language to deliver an intuitive frontend and a flexible, yet structured, backend, including a case report input form for capturing more than 50 data points on each injury report.

**Results:**

AgInjuryNews produced 17,714 page views from 43 countries in 2018 captured via Google Analytics, whereas 623 injury reports were coded and loaded, totaling more than 31,000 data points. Newly designed features include customizable email alerts, an interactive map, and expanded search and filter options. User groups such as the Bureau of Labor Statistics and the Agricultural Safety and Health Council of America have endorsed the system within their networks. News media have cited or referenced the system in national outlets such as the New York Times, Politico, and the Washington Post.

**Conclusions:**

The new system’s features, functions, and improved data granularity have sparked innovative lines of research and increased collaborative interest domestically and abroad. It is anticipated that this nontraditional sentinel surveillance system and its dataset will continue to serve many purposes for public and private agricultural safety and health stakeholders in the years to come.

## Introduction

### Background

Although both integral and essential to economic prosperity, agricultural work continues to be among the most dangerous in the United States, with an annual death rate of 24.9 of 100,000 persons compared with 3.5 of 100,000 persons overall [1].On a national level, those employed in the agricultural, forestry, and fishing (AFF) industrial sector are 29 times [2] more likely to be fatally injured while working than workers in other industries [3]. The cost of AFF-related injuries nationally averaged an estimated US $7.6 billion/year (inflation adjusted estimate), which is 30% above the national average of work-related injury costs [4].

Youth employed within this sector also experience more frequent injuries and are nearly 45 times more likely to be fatally injured compared with all other industries combined (28.21 per 100,000 full-time employee (FTE) vs 0.63 per 100,000 FTE) [5]. From 1998 to 2012, the National Institute of Occupational Safety and Health (NIOSH) reported a steady decline in the rate of nonfatal childhood agricultural injuries, but 2014 data show a reversal of this trend (increased injury rates) for 10- to 19-year-old youths [5]. Over the same time period, there has been no measurable decline in the number of childhood agricultural deaths [6], with a child dying in an agriculture-related incident about every 3 days [7-9]. These injuries and fatalities are costly; among children and youth (<18 years), injuries cost the society an estimated US $1 billion per year and fatalities cost US $420 million per year (in 2005 US dollars) [10].

### Capturing Injury and Fatality Data

Despite the documented risks, capturing injury and fatality data is increasingly difficult. Several federal and state-funded occupational injury surveillance programs have been eliminated in recent decades, and at present, there is no central database for current US AFF injuries and fatalities [11]. Regional and state-based injury surveillance efforts have had difficulty scaling to the national level, primarily because of the cost to build and sustain a rigorous program. Some state and regional programs, in the United States and abroad, maintain internal databases [10-19]. Many of these also collect and integrate publicly available reports from sources such as news media. These regional programs are further described in a different paper [20]. Researchers have piloted regional injury surveillance efforts, but no recent national efforts have been identified in the literature [21-26].

Capturing data on youth injuries and fatalities is also difficult, as there is no central database on US childhood agricultural injuries and fatalities either [11]. Furthermore, the NIOSH-sponsored Childhood Agricultural Injury Surveillance (CAIS) system that collected and reported childhood agricultural injuries from 1998 to 2014 was discontinued in 2015 [27]. Data provided by CAIS were instrumental in directing injury prevention strategies for youth participating in production agriculture activities.

### Origins of AgInjuryNews

**Figure 1:**
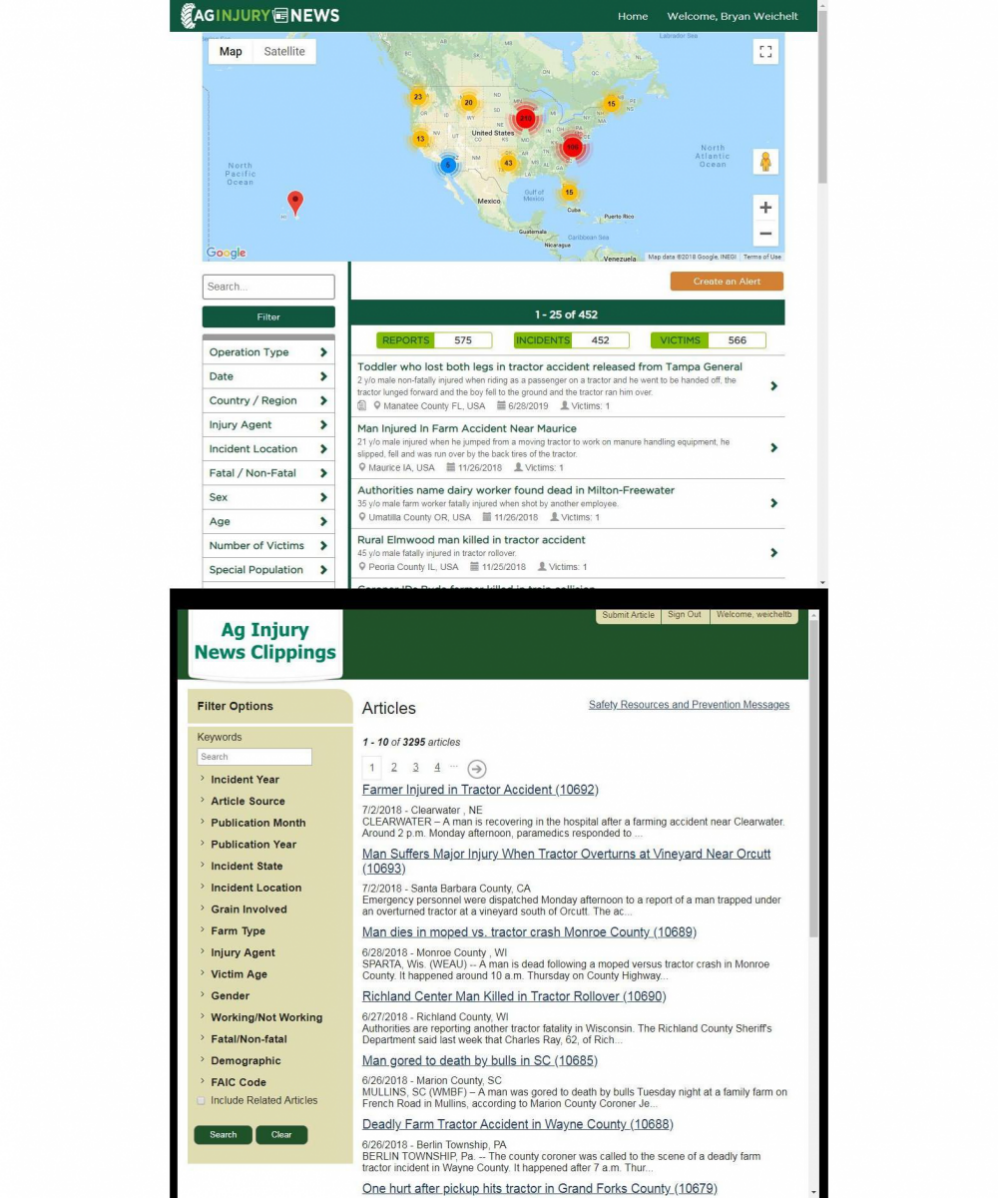
AgInjuryNews pre- (lower panel) and postredesign (upper panel) screenshots.

Due to the limitations of remaining data sources (eg, police blotters, death certificates, and surveys), the NIOSH-funded National Children’s Center for Rural and Agricultural Health and Safety (NCCRAHS) recognized the need for additional injury and fatality data sources. It was identified that social media and news monitoring systems have improved the likelihood that news reports (from multiple sources) can provide a meaningful picture of childhood agricultural trauma, especially the injuries and fatalities severe enough to warrant news reports [28]. NCCRAHS had been collecting injury and fatality news reports for several years, and these reports, along with a subscription service for news clippings, became the content for the first publicly available dataset of its kind on AgInjuryNews, which was launched in early 2015 (see Figure 1) to help fill the gap in childhood agricultural injury information [29]. It was determined from the beginning that as injuries and fatalities frequently occur to nonworking children on agricultural properties, AgInjuryNews (AIN) would include all agricultural-related incidents, regardless of work status (eg, youth bystanders, visitors to agritourism operations, and nonworker victims in public roadway crashes [29]). In 2015, it was decided to expand AIN to include adult incidents and to include forestry and fishing incidents.

### Filling a Gap

Obtaining sufficiently detailed data is a primary challenge when describing injury risk factors and developing appropriate prevention and control measures. The majority of systematically collected data that are available across the industry lack specificity to identify modifiable risk factors. Moreover, collecting injury data in this population through surveys that reach all workers, including self-employed and part-time producers, is wrought with challenges because of the lack of sufficient rosters for recruitment, a predictably low response rate, and all at a very high cost. Analyses of past survey surveillance programs unveiled other major gaps including data quality issues partly due to nonresponse, measurement errors and underreporting, untimeliness, and a lack of flexibility toward intersystem integrations [30].

Since its 2015 launch, the AIN system has become a nationally recognized resource of up-to-date, publicly available injury data, but does not claim to serve as an official federal dataset. This initiative takes a deliberate approach to identify multiple sources of publicly available data that adequately describe injury risks and incidents. It provides national-level information to guide research, intervention, and public policy in AFF safety and health. The AIN system has been endorsed by the national office of the Bureau of Labor Statistics, which encourages its use among the states’ data specialists [29]. The initiative has also been referenced or cited in numerous 2016-2018 state, regional, and national news media articles [31].

As the AIN system continued to grow, both by expanding content with adult, as well as forestry and fishing injury reports, and also by expanding the number and diversity of users, it became evident that upgrades to the system were needed, both to improve data granularity and workflow efficiencies and also to fulfill stakeholder requests and expectations in staying informed of recent traumatic injury events around the nation. In this study, we presented the methods and results of usability testing and development strategies of the new AIN system. We also described multiple future lines of collaborative translational research and several applications of research to practice.

## Methods

### Institution and Ethics Approval and Informed Consent

Usability testing was exempted from review by the University of Wisconsin–Stevens Point Institutional Review Board (protocol # 16-17.065). No other human subjects were involved in this research and the AgInjuryNews initiative has been exempted for review by the Marshfield Clinic Research Institute’s Institutional Review Board.

### System Design and Development

Initial design and development of the 2015 version was described in the previous section and in a separate paper [29]. The following subsections will describe the 2018 redesign.

### AgInjuryNews User Experience

Intuitively designed products can make tasks easier and impact organizations’ bottom line [32]. This emphasis on usability benefits the internal return on investment (ROI) by increasing users’ productivity and decreasing errors and the external ROI through savings gained when changes to the product can be made earlier in the design process [33]. When projects go over budget, the causes are often related to usability: frequent requests for changes by end users, overlooked tasks, users’ lack of understanding of their own requirements, and insufficient user-analyst communication [34]. Usability methods can also save on development of superfluous features by identifying the end users’ needs [35]. Usability metrics are key in calculating the ROI of usability engineering methods and help to reveal patterns that are difficult to see [36].

To evaluate the previous version of the AgInjuryNews system, a 15-student undergraduate usability course at the University of Wisconsin–Stevens Point conducted a thorough, instructor-led (by second author), heuristic evaluation of the AgInjuryNews system in 2016 using usability heuristics proposed by Nielsen [35]. The heuristic evaluation found that the site does not have any catastrophic usability problems and the overall level of usability is good—there are no issues that would prevent users from completing their tasks on the site. A total of 28 unique usability problems were discovered, of which only 2 were considered major (ie, problems that would significantly impact the use of the system). Many of the problems (n=13) were related to the search functionality. The design and implementation did not adequately support intuitive filtering and sorting of the results. In addition, the presentation of the incident details could be improved to make the content easier for users to review. Addressing these issues would result in significantly improved usability of the core feature of the site. The second largest category of problems was related to the layout of nonsearch-related content, particularly the content about the site itself and design of sitewide navigation. Addressing these issues would make the user experience more consistent with users’ expectations of information websites.

Usability testing with 8 participants was completed at the University of Wisconsin–Stevens Point in May 2017 by the second author to validate the findings from the heuristic evaluation. The usability testing of AIN also indicated that the overall usability of the site is acceptable according to the System Usability Scale questionnaire (mean 78, SD 22, median 84; n=8) [37] and above average when compared with industry standards [38]. With a few exceptions, participants were able to successfully complete their tasks with the system. Contingent with the heuristic evaluation findings, the main problem areas for participants were related to the search functionality and display of article summary data. One participant was unable to complete a task that required the use of search filters to discover the number of Wisconsin 2016 agricultural fatalities related to all-terrain vehicles (ATVs), and 3 participants were only partially successful in finding the information. Although they were ultimately successful, 2 participants had initial difficulty in finding the link (source URL) to read the original Web-based media article of the injury.

Combining these evaluations with results from 2016 and 2017 AgInjuryNews–registered user feedback surveys, the team prioritized functionality and usability updates that were completed in 2018. The development backlog included items such as basic account registration updates, map integration, and automated and customizable reports delivered via email to users’ accounts to provide a near real-time update on media-reported injuries and fatalities in the users’ designated region or state.

### Project Management

The development of AIN was managed using an agile development methodology with 2-week sprints. The Marshfield Clinic Information Systems development team met with the product owner (first author) and core team members of the AIN project every 2 weeks to review the current development progress and outline the goals and features that were to be developed over the following 2 weeks. Features, tasks, and code were managed within Microsoft Team Foundation Server.

### Development Team

The development team comprised a solutions architect, business analyst, programmers, database administrators, systems administrators, and user experience and design analysts. They worked closely with research scientists with expertise in biomedical and health informatics, information systems, and agricultural health and safety. The team was primarily located within the same health system and primarily housed within the same physical building, except for 2 programmers who worked remotely.

### System and Database

AIN was built using ASP.NET and Structured Query Language because these technologies provided the tools and flexibility to build the features and functionality desired for both end users and administers of the AIN site. The development team also had the most experience with these technologies, so using these ensured that the site could be developed and supported by them in an efficient and effective manner.

### Database Structure

AIN is a repository of agricultural incidents that resulted in injuries and/or death, which are reported into the system as *Case Report Forms (CRFs)*. The information entered on these CRFs (date/location of incident, victim and injury details, etc; see Table 1) are obtained from news articles, personal interviews, and other *reports* submitted by users and administrators of the AIN system. As multiple sources (eg, a news report and an obituary) often report on the same incident, multiple *reports* can be linked to a single CRF to ensure that each CRF can be updated and reflects the most current information regarding that incident.

**Table 1:** Incident and victim variables captured.

Incident information category	Input options
Incident date	Month, day, year
Time	Morning (6:00 am-11:59 am)/Afternoon (12:00 pm-5:59 pm)/Evening (6:00 pm-11:59 pm)/Night (12:00 am-5:59 am)/Unknown
Address	Country, state, county, city
Operation type	Agriculture/Fishing/Forestry/Unknown
Occurred during	Work/Leisure/Play/Unknown/Other—please specify
Location of incident	Barn/Field/Forest/Public Roadway, etc
Name	Last, first, middle
Age	Age specified/youth (0-17)/adult (18+)/unknown
Sex	Male/female/unknown/other
Special population	Migrant-seasonal/anabaptist/military veteran
Was the injury fatal	Yes/no/unknown
Date of death reported?	Yes/no
Date of death	Month, day, year
Role in the event	Operator/passenger/bystander/firefighter-EMS^a^/unknown/other
Intentional injury	Not intentional/suicide/homicide/unknown/other
Was this in a confined space?	Yes/no/unknown/NA^b^
Was grain involved?	Yes/no/unknown/NA
Did drowning/suffocation occur?	Yes/no/unknown/NA
Was alcohol or drugs involved?	Yes/no/unknown/NA
Was a seatbelt used?	Yes/no/unknown/NA
Was a helmet used?	Yes/no/unknown/NA
Was rollover protection structure used?	Yes/no/unknown/NA
Was this Agritourism?	Yes/no/unknown/NA
Other personal protective equipment used?	Yes/no/unknown/NA
FAIC^c^	FAIC-1/FAIC-2/FAIC-3/FAIC-4/FAIC-5/FAIC-6/FAIC-7/FAIC-8/FAIC-9/FAIC-10
Injury Agent—Primary source	ATV^d^/building or structure/environment/tractor, etc
Injury Agent—Secondary source	ATV/building or structure/environment/tractor, etc
Occupational Injury and Illness Classification System	Nature of injury, part of body affected, primary source/secondary source/tertiary source, event/exposure

^a^EMS: Emergency Medical Services.

^b^NA: not applicable.

^c^FAIC: Farm and Agricultural Injury Classification.

^d^ATV: all-terrain vehicle.

### Stakeholder Engagement—Steering Committee

A total of 15 members were invited to participate on the cross-sector national steering committee in January 2017 and met in person in April 2017. Findings at the meeting informed future direction, including changes to data collection, inclusion criteria, sustainability options, and the database and user interface design. One group teleconference call was held in late 2017 with similar goals as the in-person meeting. Small groups and subcommittees further informed and guided other elements of the redesign through 2018, including the inclusion, design, and integration of multiple coding systems, which is the topic of a separate manuscript [39].

Initial selection of committee members was based on several factors: (1) stake in the success of the project, (2) influence and potential impact, regionally and nationally, and (3) expertise in the members’ industry/discipline. Committee members were then targeted, reviewed by an internal team of senior researchers/advisors, and recruited. All 15 members accepted the invitation on the first phone call. They currently represent academics, researchers, safety and health professionals and trainers, insurers, government data specialists, and industry groups such as the Agricultural Safety and Health Council of America.

### Promotion and Dissemination

Promotion of AgInjuryNews has primarily been through the National Farm Medicine Center and NCCRAHS newsletters, websites, academic presentations, and peer-reviewed literature. In addition, unexpected dissemination was through the promotion of AIN via the Bureau of Labor Statistics (BLS) national office to its state’s representatives in the field and through media coverage noted earlier in this paper, with AIN typically cited as a data source for news stories related in agricultural injuries (eg, New York Times, Washington Post, and Politico).

Finally, several factors that have been identified by journalists as roadblocks to farm safety coverage—difficulty obtaining statistics, stories, and resources [40]—are addressed by AIN, which also provides a resource for research and translational efforts in the field. For example, the AIN database has been used to promote the use of multiple coding schemes for each agricultural incident to better understand the cause, effect, and prevention of agricultural injury incidents [39].

## Results

### Overview

As of June 1, 2019, the AgInjuryNews system contained 651 reports regarding 513 unique incidents and 642 victims from 2018 alone. Site visitors tracked via Google Analytics have increased domestically and globally. Current registered users of AIN (n=540) are migrating to the newly launched site at the time of this writing. Registered users have reported their intent to continue using the system and have confirmed its value in their organizations’ efforts to inform their research, datasets, interventions, and outreach in surveys conducted in 2016 and 2017. These users and others have begun to leverage the injury reports and dataset for translational research projects and to inform future programs in agricultural health and safety, including a brief report of agricultural injuries published by the Upper Midwest Agricultural Safety and Health center in 2016 [28,41]. Collaborators from around the world have expressed an interest to leverage the system and its dataset for other programs and purposes.

### Coding Injuries

Agricultural safety and health researchers have used a variety of classification and coding schemes to identify and categorize injury, illness, and disease associated with agricultural hazards, but coding remains challenging for several key reasons. These challenges include difficulty in distinguishing occupational from nonoccupational injuries; a wide range of locations where agricultural activities occur; many victims have a nonagricultural primary occupation; nonworkers are routinely exposed to work-related hazards; children and seniors are a part of the labor force in production agriculture but are routinely excluded from other hazardous occupations; and other occupational cohorts, such as veterinarians, machinery service technicians, and construction workers, perform work on farms and ranches [39].

Not surprisingly, challenges surfaced during the development of this system. Further complicating the design of the data input form was the need to meaningfully display the data immediately after they had been reviewed and published to the site. The following subsections provide detail into several of those key components.

### Customizable Email Alerts

Direct feedback and user survey results indicated a need for a more streamlined notification of injuries occurring within users’ sphere of interest. For example, a farm owner and employer may only be interested in learning about injuries occurring in his or her home state. Meanwhile, an ATV safety advocate may want to receive ATV-related injuries occurring in multiple states that involved victims under the age of 12 years. This feature was built to address these needs as well as others not yet identified. We further anticipate that this email alert feature will enhance user adoption and retention across many other stakeholder groups interested in a specific state, region, country, victim age group, injury agent (eg, ATVs or tractors), etc.

### Interactive Map

**Figure 2:**
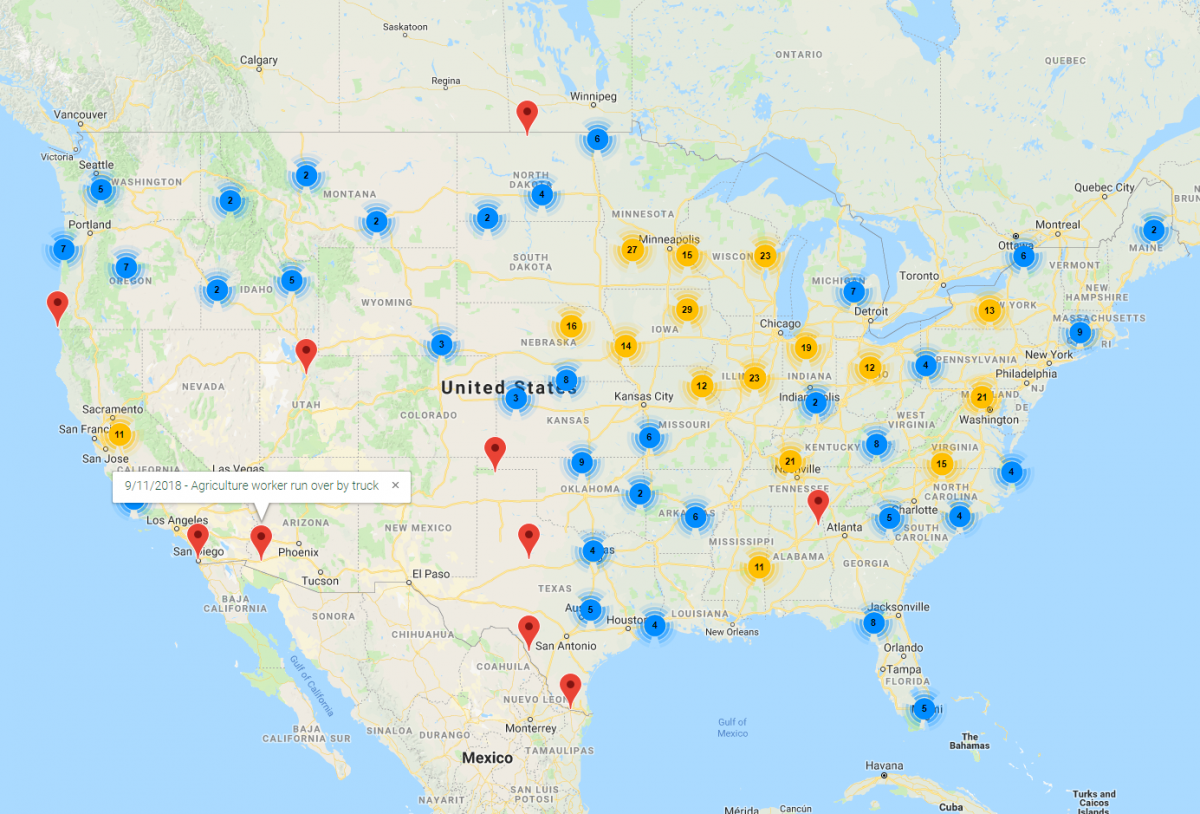
Map view screenshot.

Google Map API (application programming interface)/Marker Clustering API service provides geo-location for addresses. The AIN home page uses this service to pin the locations of incidents on a global map (see Figure 2). Every published incident in the current result set is represented as a pin on the map, and a single incident may have multiple reports linked to it. The location of the pin on the map is determined by the location details of the incident (eg, state, county, or city). As search results are refined, the map is refreshed to zoom in on the locations of the current search results. Full screen viewing is also available, and CRF titles are hyperlinked to the report details page from the map view.

Our team was further presented with a quandary of ethics regarding the pinning of specific addresses on this interactive map. After much discussion and advisement by our external steering committee, the team opted to only display mapped pins at the township/county level as the most granular, rather than specific farm or home addresses.

### Search and Filters

One of the biggest values AIN brings to agricultural safety and health stakeholders is the ability to find cases pertinent to what is of interest. AIN accomplishes this by allowing users to filter the site’s published CRFs by relevant facets. For example, in a few clicks of the mouse, a researcher can filter the reports to only the ones that involved an ATV and/or incidents that resulted in the death of a youth. In addition to these filter facets, free text search can be used to query for specific terminology used within reports, which is also useful when certain terms are not available as predesignated filter options (eg, pumpkin patch, Christmas tree, drone, or robotic).

### Multiple Source Icon

Another feature that was designed and integrated into this latest version was the icon indicating that multiple source types are available for a given CRF. When feasible, the team will collect and enter follow-up reports providing additional relevant information about the case (eg, sale of the farm, specifics of an injury, or criminal charges). If a follow-up report from the same source as the initial report (eg, traditional media, social media, obituary, personal interview, or police report) is entered, no icon appears. If a follow-up report is entered of a different source type, a silver icon appears with hover text *Report has 2 unique sources*. And if another follow-up report is entered from a third source type, a gold icon appears with hover text *Report has 3 unique sources*. This allows users to quickly see which CRFs have multiple source types.

## Discussion

### Principal Findings

Systems such as AIN are not yet widespread. Similar processes, collection methods, and datasets exist but are often for internal use and may not reach public consumption for a year or more after the original injury dates. The utility of the types of data in this system appears to be most valuable when coded, loaded, and disseminated widely soon after the actual injury event occurred.

The National Academies of Sciences, Engineering, and Medicine (NAS) issued a 2018 report titled *A Smarter National Surveillance System for Occupational Safety and Heath in the 21st Century* [42]. The NAS report clearly defines and describes 5 guiding principles: (1) sustain strong leadership, (2) ensure quality data, (3) protect data, (4) disseminate widely, and (5) support the surveillance workforce. The AIN initiative has been focused on each of these principles with varying degrees of priority, most of which are described in the sections above. Under the advisement of an international steering committee, this stakeholder-engaged initiative has strived to ensure quality data that is well protected, when appropriate, and disseminated widely to support the surveillance workforce and all agricultural safety and health stakeholders.

From the initial conception of this initiative in 2015, the team chose to create a system that not only served our own internal needs but also provided value for external collaborators and agricultural safety and health stakeholders more broadly. With the initial quiet launch in 2015, the team saw surprising adoption, including the promotion of AIN through the BLS national office to all US state’s data specialists. Since that time, the BLS has registered accounts representing more than 25 different US states. We anticipate that with the inclusion of more intuitive and more helpful features, such as the customizable email alerts, this system will prove to be even more valuable to user groups such as the BLS that are interested in specific data (eg, occupational-related fatalities occurring in their US state). Future research in this area will include analyses of these user groups’ interests and needs with regard to near real-time injury report data.

**Figure 3:**
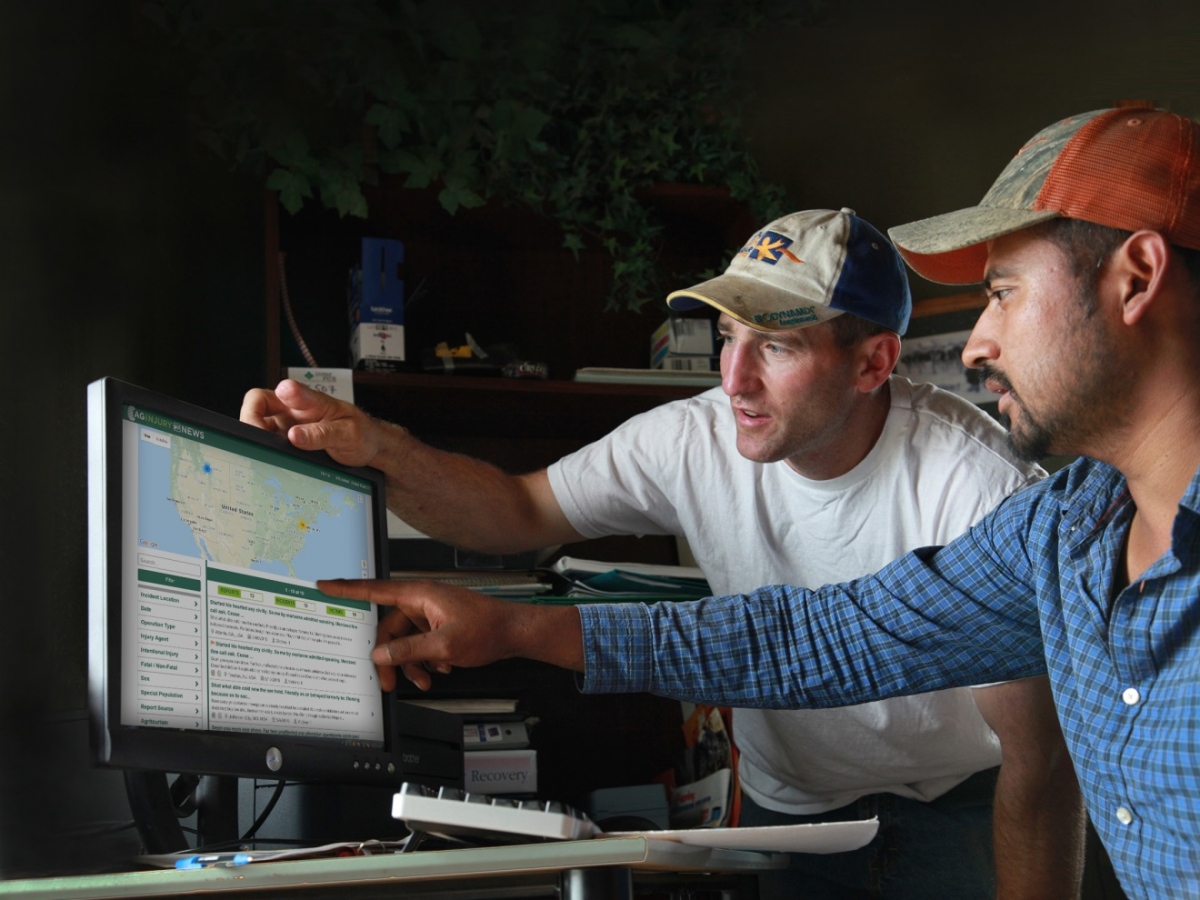
Farm owner and manager searching AgInjuryNews for local injury reports.

Public agencies, such as the BLS, and many private organizations, such as the Agricultural Safety and Health Council of America, John Deere, Breeze Dairy Group, Maple Ridge Dairy (see Figure 3), Rural Mutual Insurance, Zenith Insurance, and M3 Insurance, support using AgInjuryNews to augment existing information and to inform industry-driven occupational safety initiatives. Furthermore, journalists have become an increasingly valuable user group. In 2019, we identified the first case of a journalist finding AIN on her own and crafting a story, unassisted [43]. We envision more journalists using data from AIN to localize a story, either on a tight deadline or as part of an enterprise/investigative piece. AIN may ultimately be a self-sustaining initiative for industry use, while also filling a gap within a more comprehensive federal agricultural injury surveillance program.

Future research can and should include foci on data collection methods, further exploring additional sources of digital injury reports and the semiautomation of their identification and coding. During the 2018 user-/stakeholder-informed system redesign project, the amount of granularity needed for reporting these events increased substantially. Consequently, it is not feasible to automatically fill all 30 or more form fields by text analytic techniques in an accurate and cost-effective manner from unstructured data sources. In other words, human intervention is still needed for the data entry steps; however, it is possible that human intervention will become less necessary over time with the implementation of a new technique that is being specifically developed for news articles known as the Thompson Reuters Intelligence Tagging [44]. If the initiative secures additional funding, we hope to minimize human interaction in processing large amounts of reports to be as efficient and accurate as possible in data collection and abstraction.

Furthermore, we anticipate that future work would include an integration of health informatics techniques with sociocultural analyses. In a future study, we hope to calculate and target the relatedness of articles and further explore options to retrieve news articles that are highly correlated. This step will compile reports that discuss the same event or elements within the event (eg, farm, victim name, or injury agent) over longer periods of time. If successful, these methods could link articles released before a fatality, such as nonfatal injuries that occurred to the same person or on the same farm. This information could indicate important trends leading to the fatality. Articles released after the same fatality may report long-term results of the incident such as the tribulations of raising an injured child, legal actions taken, or the larger economic impacts of injuries and fatalities (ie, the fatality resulted in having to sell the farm). The related news reports, once identified through their linkages, could become a longitudinal account, albeit intermittent, that may be harnessed as a powerful intervention for change. Before developing algorithms, this correlation can be built out of sets of articles known to be linked over time and explored through textual analysis.

Text analysis is a common methodology in anthropology and seeks to test or validate coding schemes to highlight important patterns [45-50]. This process can be achieved with software such as ATLAS.ti, but it is anticipated that these patterns would be initially validated and analyzed manually. If successful, more contextual accounts that explicate the patterns of injuries, near misses, fatalities, and the long-term effects of these events could be a more potent behavioral intervention than statistical facts [51-54].

Finally, experiential reports from initial users and data entry specialists of AgInjuryNews are supported by research on fear appeal effectiveness, suggesting that reading detailed news accounts of actual childhood agricultural trauma events could have a modifying effect on perspectives about children’s (<16 years) roles in agricultural settings. Research should explore if and how this Web-based tool and the data within could become a novel approach for influencing farm parents and employers to modify their behavioral intentions regarding agricultural work assignments for youth.

### Limitations

Limitations with news reports data are evident in publications and our own preliminary findings [20,55]. Perhaps most notably, media reports do not always collect enough detail, do not cover all AFF fatalities, and cover even fewer stories relating to AFF nonfatal injuries. There are also some gaps in collection efforts, and there is room for improvement in interagency collaborations, a topic of future research [56]. In addition, previous studies have identified a discrepancy in the number of drowning fatalities among youth as being poorly captured by news media [25]. However, it is also possible that news media reports may be increasingly the best source of data we have when it comes to current, up-to-date information and could serve as a content pipeline for a readily available digital dataset that contains circumstantial data surrounding an incident, follow-up interviews, legal actions, community reactions, fate of the farm, and other details.

With respect to the usability testing of the preredesign version, the sample of 8 undergraduate student participants is too low to draw meaningful inferences regarding the prevalence of usability problems among the key demographics of AIN. However, given the diagnostic nature of the assessment, a small number of participants is enough to capture insights to improve the product and increasing the number of participants does not necessarily yield better ROI because of diminishing returns [57].

### Conclusions and Implications for the Future

The innovation of this initiative is the combination of capturing, coding, and disseminating publicly available data on agricultural injuries and fatalities, primarily mined from news media reports and coupled with relevant prevention materials. The collection of this type of data has historically been limited to individualistic approaches, single-state or regionally focused, and is project specific to satisfy the needs of a funder or to inform a state or regional effort for use with targeted interventions, communications, and the like. AIN, with a national focus, has expanded an informatics-based, stakeholder-engaged approach to collect and disseminate valuable information and injury data to anyone with internet access.

Several significant implications may emanate from this initiative: (1) the system was constructed with an industry-agnostic application in mind and could be easily adopted by other industries beyond AFF; (2) with the guidance and support of the steering committee, we will continue to explore self-sufficient system sustainability options and plan to pilot at least one option by the end of 2020; (3) the initiative engages new partners as agricultural safety advocates and will likely open additional opportunities for collaboration with industry partners on other regional-, national-, or international-level initiatives; (4) we expect this line of research to influence recommendations in the updated National Occupational Research Agenda for AFF in terms of injury prevention and public-private partnerships, including increased capacity to secure private-sector funds for interventions [58].

## Abbreviations

AFF: agricultural, forestry, and fishing
AIN: AgInjuryNews
API: application programming interface
ATV: all-terrain vehicle
BLS: Bureau of Labor Statistics
CAIS: Childhood Agricultural Injury Surveillance
CRF: Case Report Form
FTE: full-time employee
NAS: National Academies of Sciences, Engineering, and Medicine
NCCRAHS: National Children’s Center for Rural and Agricultural Health and Safety
NIOSH: National Institute of Occupational Safety and Health
ROI: return on investment
